# The prognostic significance of lncRNA FGD5-AS1 in various malignancies: a meta-analysis

**DOI:** 10.3389/fonc.2024.1451949

**Published:** 2024-08-19

**Authors:** Hongyan Lei, Tao Ye, Jiaxin Sun, Yongzhou Wang

**Affiliations:** Department of Gynecology, The Affiliated Traditional Chinese Medicine Hospital, Southwest Medical University, Luzhou, China

**Keywords:** LncRNA FGD5-AS1, malignancies, prognosis, biomarker, meta-analysis

## Abstract

**Background:**

Cancer is widely recognized as a prominent contributor to global mortality due to factors such as delayed diagnosis, unfavorable prognosis, and high likelihood of recurrence. FGD5 transcription factor G antisense RNA 1(FGD5-AS1), a newly identified long non-coding RNA, has emerged as a promising prognostic biomarker, for malignancy prognosis. This meta-analysis aimed to assess the prognostic significance of FGD5-AS1 in various carcinomas.

**Methods:**

A systematic search was performed through five electronic databases to identify studies that investigating the role of FGD5-AS1 expression as a prognostic factor in carcinomas. The value of FGD5-AS1 in malignancies was estimated by odds ratios (ORs) and hazard ratios (HRs) with a corresponding 95% confidence intervals (CIs). Furthermore, the GEPIA database was used to further supplement our results.

**Results:**

This analysis included 12 studies with 642 cases covering eight cancer types. High FGD5-AS1 expression exhibited a significant correlation with poor overall survival(OS) (HR = 2.04, 95%CI [1.72, 2.42], P < 0.00001), advanced tumor stage (OR = 3.47, 95%CI [2.34, 5.14], P < 0.00001), lymph node metastasis(LNM) (OR = 1.79, 95% CI [1.20,2.67], P = 0.004), and larger tumor size (OR= 5.25, 95%CI [2.68, 10.30], P < 0.00001). Furthermore, the FGD5-AS1 expression was notably upregulated in six types of malignancies as verified using the GEPIA online gene analysis tool.

**Conclusions:**

The findings of this meta-analysis indicated that high FGD5-AS1 expression was significantly associated with poor prognosis in diverse cancer types, suggesting that FGD5-AS1 may be a promising biomarker for predicting cancer prognosis.

**Systematic review registration:**

https://www.york.ac.uk/inst/crd, identifier CRD42024552582.

## Introduction

Cancer is a substantial global health concern due to escalating incidence and mortality rates, placing a substantial burden on the world economy (1). Recent statistics from the American Cancer Society reported that approximately 600,000 cancer-related fatalities in the United States in 2023, while around 1.95 million individuals were diagnosed with new cases of cancer (2). Moreover, China witnessed nearly 3 million cancer deaths and about 4.5 million new cases in 2020. Constituting approximately 30.2% of the global cancer mortality. These figures significantly surpassed those observed in the United States, thereby exacerbating the burden of cancer in China (3) Despite significant advancements in cancer surveillance, the mortality rates for many tumors have not decreased (4, 5). It has been estimated that the number of cancer cases is expected to reach 28.4 million by 2040, as indicated by recent statistics (6). Currently, cancer patients with advanced tumor stage or LNM exhibit a poor prognosis (7), yet effective cancer biomarkers for diagnosis were still scarce (8), highlighting the urgent need for exploring novel biological targets in early detection and prognosis prediction.

RNAs of greater than 200 nucleotides length (lncRNAs) play a crucial role in various biological processes, including the regulation of gene expression and the modulation of nuclear architecture (9). Recently, accumulated evidence suggests a strong association between lncRNAs and prognosis in various malignancies, revealing such targets have great potential to detect and treatment of cancer (10, 11). Multiple studies have indicated that FGD5-AS1 is significantly elevated in different cancerous tissues and has a close correlation with metastasis, tumor staging, tumor size, and survival time (12, 13). FGD5-AS1 is a novel lncRNA, which associated with tumorigenesis and tumor development (14). Collectively, FGD5-AS1 represents a highly promising biomarker for cancer prognosis.

FGD5-AS1, a recently characterized lncRNA, has been observed in diverse carcinomas, including lung cancer (13, 15, 16), ovarian cancer (17) gastric cancer (GC) (18, 19), melanoma (20), hepatocellular carcinoma (21), glioblastoma carcinoma (22, 23), osteosarcoma carcinoma (24), and breast cancers (25), From a clinical perspective, there was a strong correlation observed between elevated levels of FGD5-AS1 and poor OS as well as clinicopathological features for example progressed tumor stage, presence of LNM, and larger tumor size (17, 24). Additionally, FGD5-AS1, strongly associated with m6A regulators, emerges as a promising biomarker and potential drug targets (26). However, because of the small number of patients in the clinical sample, the impact of this connection is not well defined. Therefore, we performed this meta-analysis to assess the prognostic significance of FGD5-AS1 as a biomarker in different types of cancers.

## Materials and methods

This study was conducted following the Preferred Reporting Items for Systematic Reviews and Meta-analyses (PRISMA) (27).

### Search strategy

The databases listed below were comprehensively searched from their inception until January 10, 2024: Web of Science, Springer, Cochrane Library, PubMed, and EBSCO. We use the following keywords to identify relevant publications: FGD5-AS1 anti-sense RNA 1, long non-coding RNA FGD5-AS1, lncRNA FGD5-AS1, and FGD5-AS1. Additionally, the references in these retrieved studies were estimated carefully to identify more potential relevant records.

### Inclusion and exclusion criteria

The analysis would include studies that meet the following inclusion criteria: (I) The present study investigated the correlations between FGD5-AS1 expression and various human malignancies; (II) Subjects were categorized into two groups according to their FGD5-AS1 expression levels: high and low expression groups; (III) The study reported clinicopathological parameters, OS or disease-free survival (DFS) data; (IV) No preoperative chemotherapy or radiotherapy was administered to the patients; (V) the study samples were derived from tissue; (VI) there are no ethnic and geographical restrictions.

Exclusion criteria include: (I) Data obtained from public databases; (II) Studies without clinicopathologic parameters, OS, or DFS; (III) Based on cells, reviews, non-tumor studies, or retracted articles.

### Data extraction and quality assessment

After conducting a comprehensive examination of all eligible articles, the salient features of the eligible studies were independently extracted by the first two authors. The recorded items included: author’ s last name, types of cancer, year of publication, the capacity of sample size, the level of FGD5-AS1 expression in patients, technique used for detection, the HR and its 95% CI were also assessed for OS, follow-up time and extraction methods for OS data. If Kaplan-Meier curves were not provided alongside HR and 95% CI values, using Engauge Digitizer 4.1 software, the HR (95% CI) was indirectly calculated for OS (28). Any discrepancies should be resolved by timely discussion with the corresponding author.

### Validation of FGD5-AS1 expression in bioinformatics database

We compared the differential expression of FGD5-AS1 between normal tissues and various tumor tissues. Furthermore, we evaluated the prognostic significance of gene expression profiling interactive analysis (GEPIA) by utilizing publicly accessible data from TCGA and GTEx databases (29–33). The correlation between FGD5-AS1 expression and DFS or OS was assessed across cancer datasets, revealing its significant impact on patient outcomes. Statistical significance was determined for all P < 0.01.

### Statistical analysis

Statistical analysis was performed using Review Manager 5.3 software. We conducted a comprehensive analysis to evaluate the correlation between FGD5-AS1 expression and clinicopathological characteristics, utilizing combined OR and 95% CIs. Furthermore, we analyzed to assess the association between the expression of FGD5-AS1 and OS in different types of malignancies by calculating ORs and HRs with 95% CIs. If I^2^ ≤ 50%, the fixed-effects model was utilized. In other cases, the random effects model was applied. A statistically significant value of P < 0.05 was considered. A sensitivity analysis was carried out by omitting individual papers to investigate the robustness of the pooled results, if five articles (at least) were included.

## Results

### Included articles

**Figure 1:**
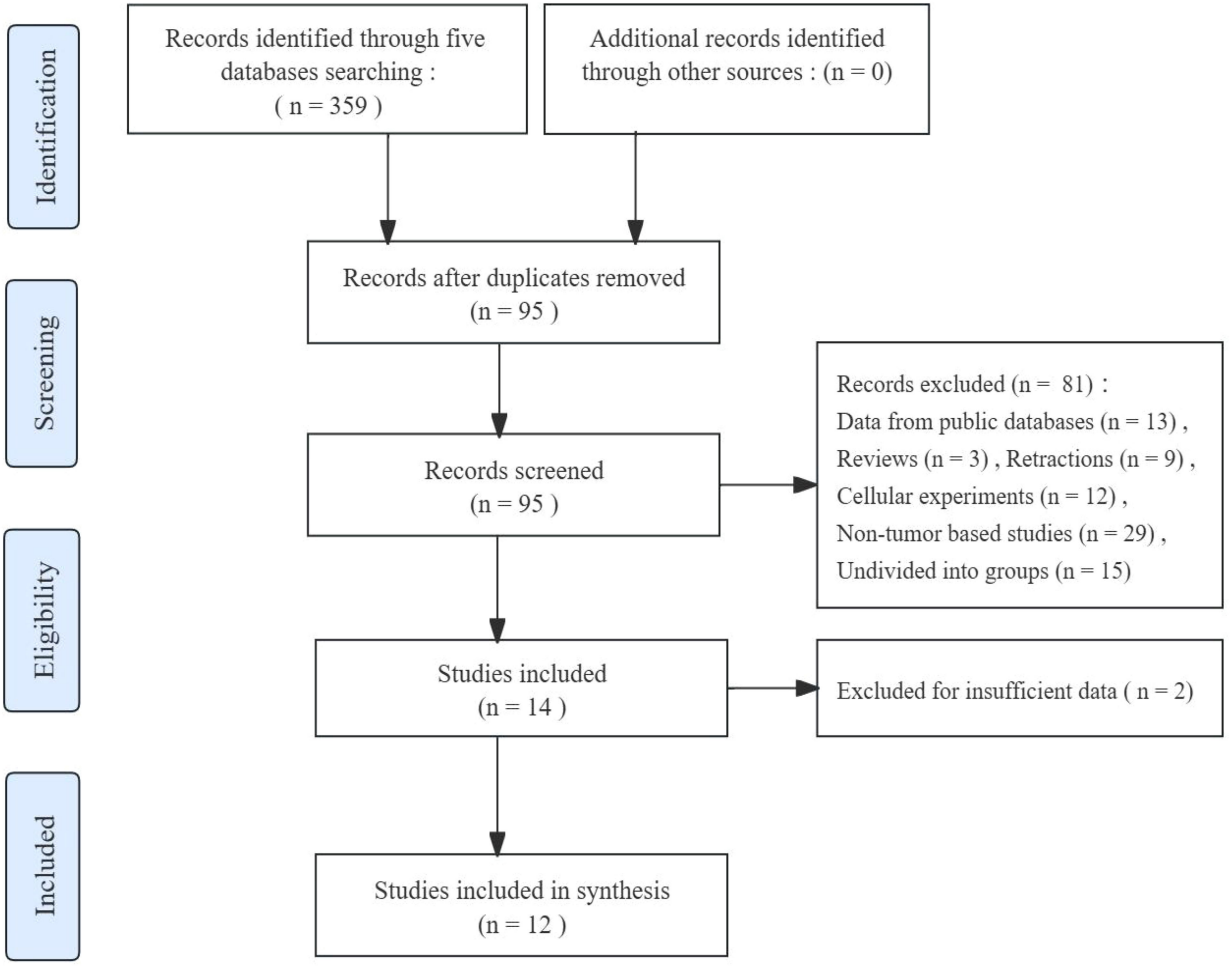
Article selection flowchart.

The article selection process is depicted in **Figure 1**. After conducting an initial screening search across five databases, a total of 359 records about the association between FGD5-AS1 and cancer prognosis were obtained. Subsequently, 264 duplicate publications were removed, the remaining 95 studies for further evaluation. Additional exclusion criteria were applied to these studies resulting in their elimination (81 studies excluded). Following full-text screening, two records with insufficient data were also excluded. In the end, a meta-analysis was conducted using a compilation of 12 published studies.

### Study characteristics

A meta-analysis was conducted, encompassing 12 studies involving 642 cancer patients from China. Quantitative real-time polymerase chain reaction (qRT-PCR) was utilized to detect FGD5-AS1 expression in tissues. The sample size ranged from 30 to 188 individuals, with a distribution of 323 cases exhibiting high FGD5-AS1 expression and 319 cases showing low FGD5-AS1 expression. In addition, all the studies included in this analysis presented clinicopathological characteristics including lymph node involvement, patient age, tumor dimensions, sex distribution, tumor grade, and disease stage. Among the included studies, seven reported OS, while one reported DFS. This meta-analysis encompassed eight different types of malignant tumors. **Table 1** provides a comprehensive overview of the specific attributes found in these studies that have been incorporated.

**Table 1:** Main characteristics of included studies.

Author	Year	Malignancy types	Sample sizes (*n*)	Detection method	Outcomes	HR (95% CI) for OS	Follow up (*m*)	Data extraction method
Wu (22)	2020	Glioblastoma carcinoma	64	qRT-PCR	OS	1.93 (0.91, 4.1)	80	indirectly
Su (23)	2020	Glioblastoma carcinoma	30	qRT-PCR	CP	NA	NA	NA
Fan (15)	2020	Non-small cell lung carcinoma	50	qRT-PCR	CP, OS	2.33 (1.01, 5.37)	60	indirectly
Song (24)	2020	Osteosarcoma	97	qRT-PCR	CP, OS	1.68(0.65, 3.31)	60	indirectly
Gao (20)	2020	Melanoma carcinoma	188	qRT-PCR	CP, OS	2.986(1.218, 4.652)	60	directly
Lv (13)	2021	Non-small cell lung carcinoma	65	qRT-PCR	CP	NA	NA	NA
Fang (16)	2021	Non-small cell lung carcinoma	35	qRT-PCR	OS	2.02 (0.66, 6.14)	60	indirectly
Liu (19)	2021	Gastric carcinoma	30	qRT-PCR	CP	NA	NA	NA
Li (25)	2021	Breast carcinoma	50	qRT-PCR	CP	NA	NA	NA
Feng (18)	2022	Gastric carcinoma	66	qRT-PCR	OS	1.59(0.65, 3.91)	80	indirectly

CP, clinicopathologic parameters; OS, overall survival; NA, not applicable.

### Association between the expression of FGD5-AS1 and clinical covariates

**Figure 2:**
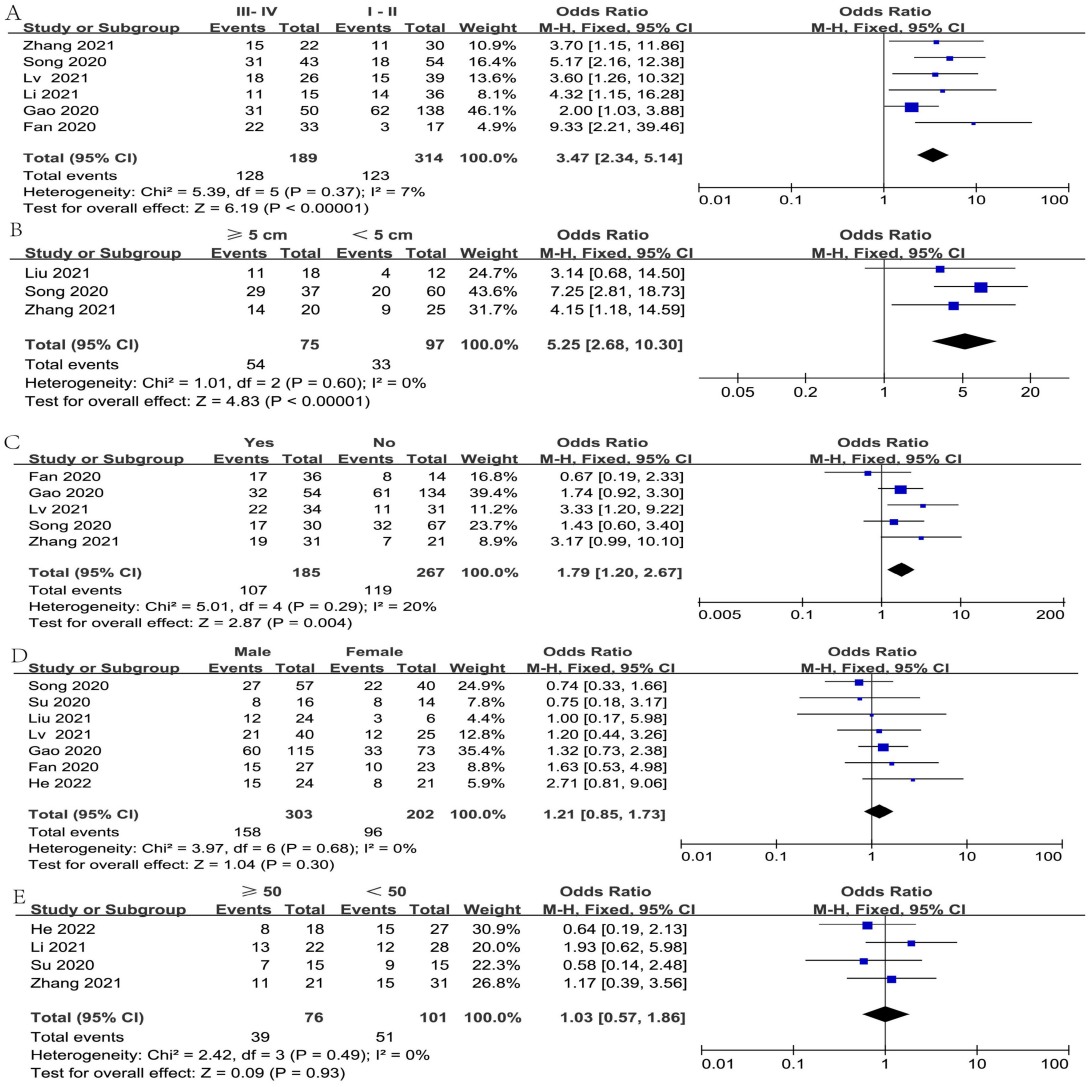
The objective of constructing forest plots was to estimate the correlation between FGD5-AS1 expression and clinicopathological factors, for example tumor stage **(A)**, tumor size **(B)**, LNM **(C)**, gender **(D)**, and age **(E)**.

The relationship between FGD5-AS1 and clinical covariates was investigated in the meta-analysis, as depicted in **Figure 2**. The covariate of gender was not considered in the analysis due to the exclusion of two studies (cases exclusively involving females). Since all included studies were carried out in different hospitals, the cut-off values for the age of patients and tumor sizes were not uniform. Hence, we considered patient age < 50 years and ≥ 50 years, along with tumor sizes ≥5 cm and < 5 cm as our analytical data, which were adopted by most of the included studies.

After omitting one study (19) via sensitivity analysis, the heterogeneity diminished from 62% to 7%. The result from six articles illustrated a significant association between high FGD5-AS1 expression and tumor stage (I^2^ = 7%, P < 0.00001) (**Figure 2A**). Besides, Furthermore, four studies assessed the correlation of FGD5-AS1 expression with tumor size; after excluding Su’s study (23) by sensitivity analysis, the heterogeneity reduced from 56% to 0%; hence, the pooled results revealed that high FGD5-AS1 expression was significantly associated with larger tumor size (I^2^ = 0%, P < 0.00001) (**Figure 2B**). Furthermore, the pooled results also revealed that high FGD5-AS1 was considerably correlation advanced lymph node metastasis (I^2^ = 20%, P *=* 0.004)(**Figure 2C**). However, FGD5-AS1 expression was not evidently associated with gender (I^2^ = 0%, P = 0.30)(**Figure 2D**) and age (I^2^ = 0%, P = 0.93) (**Figure 2E**).

### Association between the expression of FGD5-AS1 and OS

**Figure 3:**
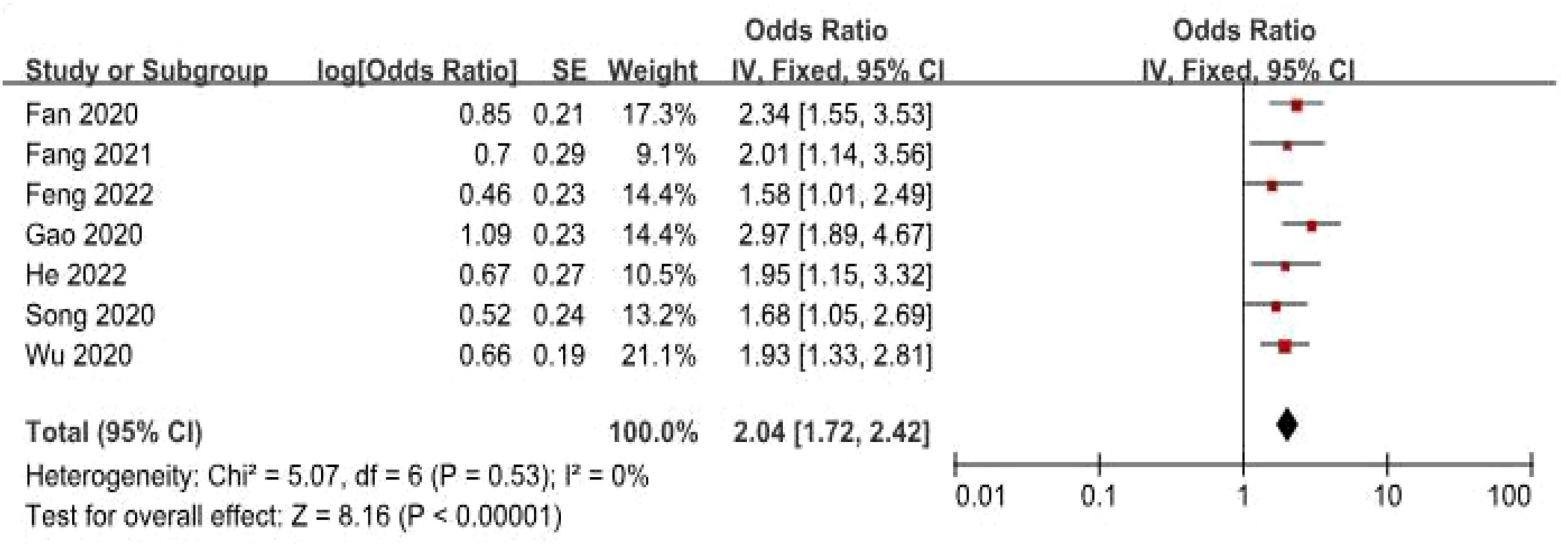
The forest plot illustrates the correlation between FGD5-AS1 expression levels and OS.

Data from seven studies, which included 545 patients, were analyzed to investigate the association between FGD5-AS1 as a predictive indicator for cancerous conditions and OS. The pooled HR was calculated as 2.04 (95% CI [1.72, 2.42], P < 0.00001). To evaluate the hazard ratio of these studies, a fixed-effect model was used as there was minimal heterogeneity (I^2^ = 0%). The findings indicated that upregulation of FGD5-AS1 predicts unfavorable OS across diverse carcinomas (**Figure 3**). Additionally, subgroup analysis was conducted based on cancer types and follow-up time. According to the data presented in **Table 2**, cancer patients with higher expression levels of FGD5-AS1 showed a significant association with poor OS results, irrespective of cancer type and follow-up duration (P < 0.00001). To account for minimal or negligible heterogeneity observed in all stratified analyses, a fixed-effect model was utilized.

**Table 2:** The forest plot demonstrates the correlation between FGD5-AS1 expression levels and OS in a visually informative manner.

Variables	Total cases (*n*)	HR 95% CI	P	I^2^ (%)	Model
Cancer type					
Gastrointestinal cancer	111	1.73 [1.23, 2.44]	P = 0.002	0	Fixed
Respiratory cancer	85	2.22 [1.59, 3.10]	< 0.00001	0	Fixed
Others cancer	349	2.12 [1.66, 2.70]	< 0.00001	40	Fixed
Follow-up time (*m*)					
> 60	110	1.78 [1.34, 2.38]	< 0.0001	0	Fixed
≤ 60	415	2.19 [1.77, 2.71]	< 0.00001	0	Fixed

### Validation of the findings using the TCGA dataset

**Figure 4:**
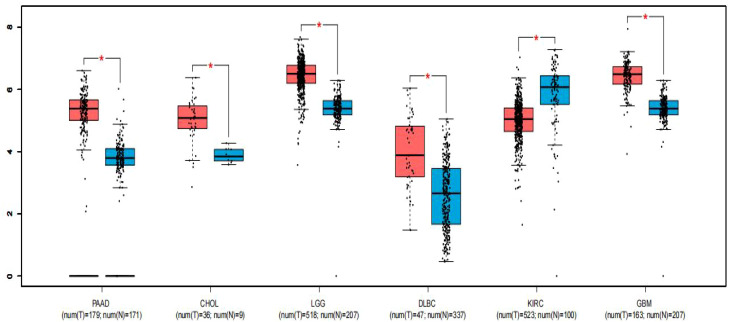
Expression of FGD5-AS1 in six types of tumor tissues (depicted in red) compared to normal tissues (depicted in blue). Statistical significance denoted by “*” indicates P < 0.01. T, tumor tissues; N, normal tissues.

To strengthen the reliability of our results, we utilized the GEPIA online gene analysis tool to survey the expression profile of FGD5-AS1 in various malignancies. Notably, **Figure 4** demonstrates significant upregulation of FGD5-AS1 in six various categories of cancers (P < 0.01), including pancreatic adenocarcinoma (PAAD), cholangiocarcinoma (CHOL), lower-grade glioma (LGG) of the brain, diffuse large B-cell lymphoma (DLBCL), glioblastoma multiforme (GBM), and clear cell renal carcinoma of the kidney (KIRC).

## Discussion

Recently, an increasing body of research has revealed the dysregulation of lncRNAs in various human malignancies, suggesting their potential as novel biomarkers for early cancer diagnosis, therapy selection, and prognosis prediction. Consequently, recent years, a multitude of articles have been conducted to progressively elucidate the intricate association between lncRNAs, and diverse tumor prognoses (34–36). FGD5-AS1, a recently discovered lncRNA, has gained significant attention as a crucial oncogene due to its strong association with adverse clinicopathologic characteristics and unfavorable prognosis among individuals diagnosed with cancer (12). The intricate mechanisms underlying the cellular functions associated with its involvement in carcinomas have not been thoroughly investigated through a meta-analysis to assess the prognostic significance of FGD5-AS1 expression in individuals diagnosed with cancer.

In addition, the underlying mechanisms governing the expression of FGD5-AS1 in malignancies exhibit a high degree of complexity. Zhang, et al. found that the expression of FGD5-AS1 was upregulated in hepatocellular carcinoma (HCC) tissues (37). Li, et al. observed that FGD5-AS1 promotes osteosarcoma (OSA) cell proliferation and migration by acting as a miR-506-3p sponge, thereby upregulating RAB3D (38). Furthermore, Song, et al. demonstrated that overexpression of lncRNA FGD5-AS1 is associated with cisplatin resistance in LSCC (39). Xu, et al. identified the correlation between m6A-related lncRNA FGD5-AS1 and cancer treatment, particularly how it affects the development of resistance to cisplatin in individuals with breast cancer (26). Collectively, these compelling pieces of evidence highlight the pivotal role played by FGD5-AS1 in tumor advancement and growth.

In this meta-analysis, we observed a noteworthy correlation between FGD5-AS1 expression and prognostic factors in human malignancies. The results demonstrated that increased levels of FGD5-AS1 were strongly associated with unfavorable OS (HR = 2.04, 95%CI [1.72,2.42], P < 0.00001). Subgroup analysis further confirmed these findings across different cancer types and follow-up durations. Moreover, high expression of FGD5-AS1 was significantly associated with tumor stage (OR = 3.47, 95% CI [2.34,5.14] P < 0.00001), lymph node metastasis (OR = 1.79, 95% CI [1.20,2.67], P = 0.004), and larger tumor size (OR= 5.25, 95%CI [2.68, 10.30], P < 0.00001) in cancer patients. No noticeable heterogeneity or publication bias was detected among the studies included. Additionally, analysis conducted using the GEPIA online database revealed that increased expression of FGD5-AS1 served as a prognostic indicator for unfavorable OS in six distinct types of cancer. However, no notable association between FGD5-AS1 expression and gender or age was identified. In addition, to strengthen the reliability of our results, we utilized the GEPIA database extensively and identified elevated expression of FGD5-AS1 in six various types of cancers. Notably, six cancer types including PAAD, CHOL, LGG, GBM, DLBC, and KIRC lacked previous studies investigating the significance of FGD5-AS1 with cancer prognosis. Overall, our results strongly suggest that FGD5-AS1 has potential as a novel prognostic biomarker for various malignancies and warrants further investigations into its association with cancer prognosis.

However, there are some limitations to the study. First, despite not imposing ethnic and geographical restrictions during our record review process, the studies included in this analysis were exclusively conducted within the research context of China. Consequently, the generalizability of the relevant findings extended to regions outside of China may be somewhat limited. Second, in certain studies, only Kaplan-Meier curves were provided for OS, necessitating the indirect extraction of HR and 95% CIs using Engauge Digitizer 4.1 software introduced by Tierney, et al (28). To avoid subjective bias, HR and 95% CI values were extracted from Kaplan-Meier curves by two independent reviewers, and the final data came from the average of two independent data. Fourth, although DFS is a crucial concern for cancer patients, only one study included in our analysis investigated the association between FGD5-AS1 and DFS. Furthermore, we were unable to fully evaluate their relationship, which may be considered a limitation of this study. To overcome these limitations, conducting additional high-quality multicenter research is essential to further confirm the clinical value of FGD5-AS1 as a cancer prognostic indicator.

Recommendations for future research: First, efforts should be made to standardize study designs and methodologies to reduce heterogeneity and improve the reliability of meta-analyses. Second, researchers should aim to report HR and 95% CI values directly rather than relying on indirect extraction methods. Lastly, focus on additional prognostic endpoints: more studies should investigate the relationship between FGD5-AS1 and other important prognostic endpoints, such as DFS, to provide a more comprehensive understanding of its prognostic value.

## Conclusion

In summary, our comprehensive analysis reveals that FGD5-AS1 is significantly overexpressed in diverse carcinomas. Furthermore, high FGD5-AS1 expression is strongly associated with advanced tumor-stage, LNM, and poor OS compared to low expression levels. The potential of FGD5-AS1 as a biomarker for advancing cancer management underscores its promising role and significance in oncology.

## Data Availability

The datasets presented in this study can be found in online repositories. The names of the repository/repositories and accession number(s) can be found in the article/supplementary material.
